# Variation in 10 year birth trajectories of mothers involved in family court care proceedings and their peers in England.

**DOI:** 10.23889/ijpds.v9i5.2641

**Published:** 2024-09-18

**Authors:** Ruth Gilbert, Georgina Ireland, Matthew Jay, Linda Wijlaars

**Affiliations:** 1 GOSH Institute of Child Health, University College London

## Objective

Family court (FC) care proceedings decide on removing children from parental care in response to concerns about risks of harm. Courts and social care report frequent births, often after short birth intervals as a key problem among mothers presenting to FCs.

## Approach

We used a cohort of mothers with a first live birth (FLB) between 2007 and 2019 to estimate the cumulative incidence of FC within 10 years and compare birth trajectories with peers.

## Results

3,118,327 women (15-50 years) had a FLB, of whom 34,062 had FC involvement. Cumulative incidence of FC within 10 years from FLB was 1.3% (95% Confidence Interval [CI]; 1.3-1.3) overall but highest in mothers who were younger (6.7% if <20 years), more deprived (2.5% in most deprived areas) and with morbidity (22.7% with learning or multiple disability, 12.8% with adversity-related injuries or 5.7% with mental health admissions).

If followed for 10 years from FLB, 58.4% (8,889/15,229) of women with FC involvement had ≤2 deliveries, compared to 82.2% otherwise (1,007,365/1,225,163). If <25years at FLB, women involved in FC had more deliveries than their peers (3+ deliveries: 47.8%, 5,853/12,255 vs 29.5%, 123,092/416,904), however, if 25+, they had fewer deliveries if involved in FC (1 delivery: 49.6% 1,475/2,974 vs 39.2% 316,976/808,259).

## Conclusions and Implications

At-risk mothers may benefit from early, targeted health and social care interventions to prevent FC involvement. Birth data highlights variation in birth trajectories and dispels the stereotype of women involved in FCs having many children, particularly if older at FLB.

